# Imprint of the Atlantic Multidecadal Oscillation on Tree-Ring Widths in Northeastern Asia since 1568

**DOI:** 10.1371/journal.pone.0022740

**Published:** 2011-07-27

**Authors:** Xiaochun Wang, Peter M. Brown, Yanni Zhang, Laiping Song

**Affiliations:** 1 College of Forestry, Northeast Forestry University, Harbin, Heilongjiang, China; 2 Rocky Mountain Tree-Ring Research, Fort Collins, Colorado, United States of America; 3 College of Landscape Architecture, Northeast Forestry University, Harbin, Heilongjiang, China; Universidade de Vigo, Spain

## Abstract

We present a new tree-ring reconstruction of the Atlantic Multidecadal Oscillation (AMO) spanning 1568–2007 CE from northeast Asia. Comparison of the instrumental AMO index, an existing tree-ring based AMO reconstruction, and this new record show strongly similar annual to multidecadal patterns of variation over the last 440 years. Warm phases of the AMO are related to increases in growth of Scots pine trees and moisture availability in northeast China and central eastern Siberia. Multi-tape method (MTM) and cross-wavelet analyses indicate that robust multidecadal (∼64–128 years) variability is present throughout the new proxy record. Our results have important implications concerning the influence of North Atlantic sea surface temperatures on East Asian climate, and provide support for the possibility of an AMO signature on global multidecadal climate variability.

## Introduction

The Atlantic Multidecadal Oscillation (AMO) is a mode of natural climate variability occurring in the North Atlantic Ocean. AMO has a principal expression in mean sea surface temperatures (SST) anomalies north of the equator with a period of 65–80 years and an amplitude of ∼0.4°C on a multidecadal timescale [1], [2]. The AMO is thought to be induced by Atlantic Meridional Overturning Circulation (AMOC) variations and associated ocean heat transport fluctuations [1], [3]. It exerts significant influences on global and regional climates [2], [3], such as North American and European summer rainfall regimes [4]–[6], African [7], [8] and northeastern Brazilian rainfall patterns [9], [10], and the frequency of severe Atlantic hurricanes [7]. In addition, the AMO alternately obscures and exaggerates the global increase in temperatures due to human-induced global warming [11], [12].

Although AMO is a feature of the North Atlantic Ocean basin, recent studies suggest that it is also related to multidecadal variability of Asian and Indian monsoons [13]–[17]. Through comprehensive observational analyses and ensemble experiments with atmospheric general circulation models (AGCMs), it was found that twarm-phase AMO leads to warmer winters in much of China, resulting in less precipitation in coastal areas of southern China and more precipitation in northern China [17]. Wang et al. [16] extended these analyses to examine the seasonal dependence of the AMO influence on Asian monsoon. Their results indicated that warm-phase AMO causes increases in air temperature in East Asia and rainfall in Northeast China in all four seasons. In addition, positive phases of AMO induce strong Southeast and East Asian summer monsoons, and a late withdrawal of the Indian summer monsoon [15]. All these studies have together demonstrated a probable influence of Atlantic SST anomalies on Asian climate on multidecadal timescales.

However, only two full cycles of the AMO (from warm to cool phases) are represented in instrumental records. Thus, it is difficult to fully understand the low-frequency characteristics of AMO and mechanisms of its longer-term influence on East Asian climate [18], [19].

Several high-resolution proxies have been developed and used to assess longer-term annual to multidecadal variability in areas surrounding the North Atlantic, but none have looked at the longer-term (multicentennial) characteristics of AMO in East Asia [1], [19]–[21]. In this study, a climate signal with consistent AMO properties has been identified in Scots pine (*Pinus sylvestris*) tree-ring widths from trees growing in central eastern Siberia and northeastern China over the past 440 years (1568–2007 CE). This proxy reconstruction suggests a secular influence of AMO on moisture availability in the region. Perhaps most importantly, it provides support to the potential global nature of multidecadal climate variability related to AMO. The reconstruction is compared with both the instrumental AMO record and an existing multicentennial tree-ring reconstruction of AMO [20] to evaluate the AMO signal reliability.

## Methods

**Figure 1:**
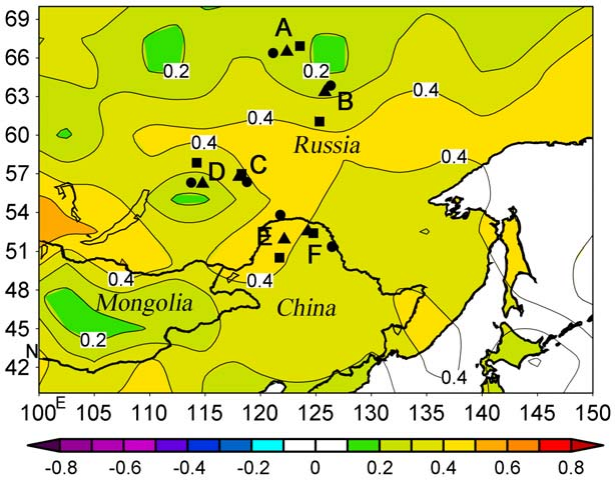
Map of correlation between annual mean precipitable water and annual AMO index (1948–2007) across northeast Asia. Country boundaries for Russia, Mongolia, and northeast China are shown on the map. It is plotted by the NOAA/ESRL Physical Science Division, Boulder Colorado (http://www.esrl.noaa.gov). Letters on the map represent different tree-ring sampling sites: **A** – Zhigansk, **B** –Khotugn, **C** – Tschita, **D** – Taksimo, **E** – Mangui, **F** – Mengkeshan. Triangles, circles, and squares represent sampling sites, PDSI points, and weather stations, respectively. Differents colors represent different correlation coefficients marked as the legend at the bottom of the map. The figure on a contour represents the correlation coefficient of this contour.

A total of 191 tree-ring width records from moisture-sensitive Scots pine from six localities in northeastern Asia were assembled for this analysis (Fig. 1, Table 1). Four chronologies from Zhigansk, Khotugn, Tschita and Taksimo in central Siberia, Russia [22] were obtained from the International Tree Ring Data Bank (ITRDB; http://www.ncdc.noaa.gov/paleo/treering.html). Two new chronologies were generated from 72 individual Scots pine trees from Mangui and Mengkeshan in northeastern China. To preserve the low-frequency variability, all raw tree-ring series were standardized by fitting them to negative exponential curves or linear regressions with negative or zero slopes. A nested approach as described in [23], which takes into account the decreasing number of chronologies back in time, was used to develop the longest possible proxy record. The first principle component of the chronologies (PC1) was employed to compare with the instrumental and Gray et al. (2004) tree-ring based AMO records. The standard chronologies were used in all the following analysis.

**Table 1:** Site information and general statistics of six Scots pine tree-ring chronologies in northeast Asia.

Site name	Taksimo	Tschita	Mangui	Khotugn	Mengkeshan	Zhigansk
Country	Russia	Russia	China	Russia	China	Russia
Latitude (N)	56°20′	56°50′	52°03′	63°23′	52°37′	66°31′
Longitude (E)	114°40′	118°05′	122°06′	125°48′	124°18′	122°20′
Altitude (m)	510	700	714	100	720	180
Time span	1707–1996	1720–1996	1791–2008	1568–1991	1628–2007	1564–1991
Tree number	32	28	34	28	38	31
MS	0.14	0.16	0.15	0.19	0.13	0.20
SD	0.17	0.20	0.27	0.26	0.28	0.23
AC1	0.49	0.48	0.75	0.59	0.87	0.37
MC	0.34	0.45	0.30	0.43	0.21	0.34
SNR	13.5	13.0	11.5	14.9	8.0	12.1
EPS	0.93	0.93	0.92	0.94	0.89	0.92
VFE %	37.9	49.8	35.6	46.9	29.7	37.4

Notes: MS-Mean sensitivity; SD-Standard deviation; AC1-Autocorrelation order 1; MC-Mean correlation; SNR-Signal-to-noise ratio; EPS-Expressed population signal; VFE-Variance in first eigenvector.

Correlations between the six chronologies and the monthly instrumental AMO index calculated from North Atlantic SST anomalies [4], [24] were determined using Pearson correlation coefficients. Palmer Drought Severity Indices (PDSI) during warm (1930–1960) and cold (1970–1990) AMO phases [20] from six nearby climate stations also was compared to the PC1 series to assess the impact of AMO on local moisture conditions. PDSI is a prominent and often used index of meteorological drought to measure departures from local mean moisture conditions [25]. To identify the major periodicities present in our PC1 proxy record, a multi-tape method (MTM) spectral analysis [26] was performed. In addition, a cross wavelet analysis was employed to determine common power and relative phases of our PC1 record and the instrumental and reconstructed AMO [20] records in the time-frequency domain [27]. The Morlet wavelet (with ω_0_ = 6) was used in this analysis and the wavelet power significance was tested at the 95% confidence level against a red-noise background [28].

## Results

**Figure 2:**
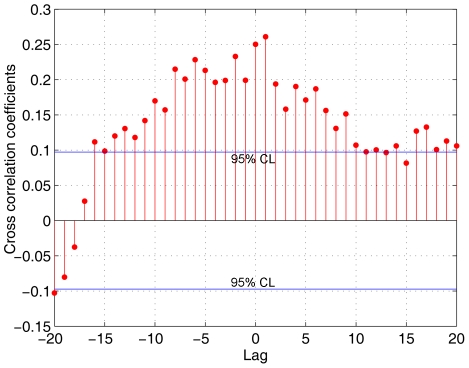
Cross correlation between the tree-ring record from northeast Asia and Gray et al. (2004) AMO reconstruction. Blue horizontal line represents a 95% significance level tested by Pearson correlation analysis.

**Figure 3:**
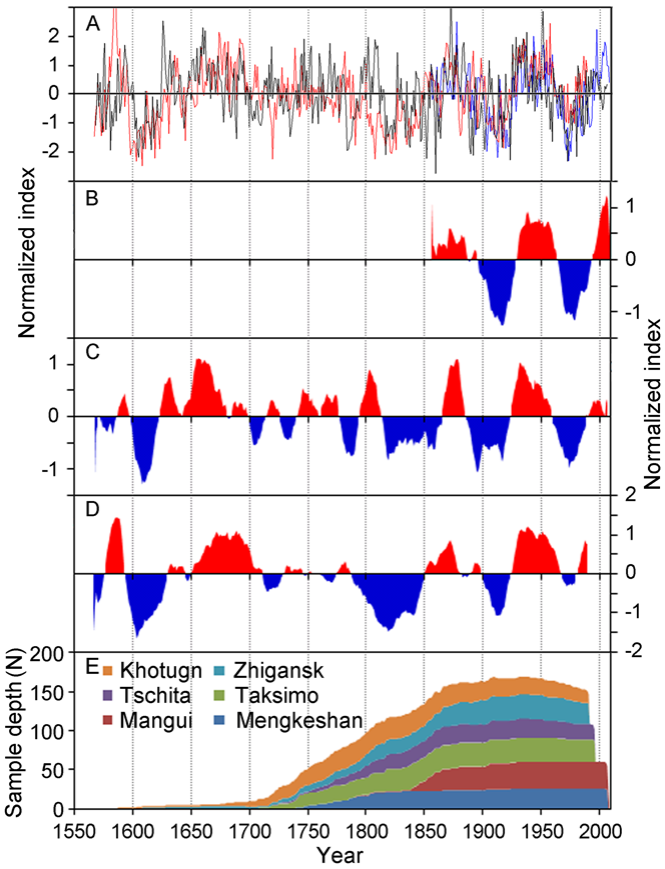
Comparisons of the intrumental, reconstructed (this study) and Gry et al. (2004) reconstructed AMO index on annual and 11-year moving average basis. (**A**) Annual comparison of instrumental AMO index [4] (blue line), the reconstructed proxy series from this study (black line), and [20] tree-ring based AMO reconstruction (red line). (**B–D**) The above three records smoothed with an 11-year low-pass filter. Red and blue shaded areas represent warm and cold AMO phases respectively. All series (A–D) were normalized by their means and standard deviations. (**E**) Sample depth in number of cores for the six tree-ring width chronologies.

Correlation coefficients between the instrumental AMO series and individual tree-ring chronologies used to develop the new reconstruction show weak (Zhigansk), moderate (Khotugn and Mengkeshan), and strong (Taksimo, Tschita and Mangui) responses of tree growth to long term changes in North Atlantic SST (Table 2). PC1 of the six East Asian chronologies exhibits correlations of 0.37 and 0.70 with the annual and 11-year moving average AMO instrumental index (1856–2007), and correlations of 0.25 (Fig. 2) and 0.44 with the annual and 11-year moving average of the tree-ring based AMO reconstruction (1568–1990) [20], separately. The East Asian PC1 series, the AMO instrumental index, and the existing tree-ring based AMO record from the Atlantic rim exhibit similar annual and multidecadal patterns of variation over the past 152 years (Fig. 3). Furthermore, the AMO warm and cool phases of the East Asian series and the reconstruction from the Atlantic rim exhibit good correspondence except for the 18^th^ century over the last 423 years (Fig. 3). The interval from the late1700s to the early 1800s seems to be a quasi-quiescent period of the AMO, which does not differ significantly from the series mean [20]. These results suggest that the variability observed in the instrumental AMO series imprint on tree-ring widths and that the warm AMO phases increase radial growth of Scots pine forests in northeast Asia.

**Table 2:** Correlation coefficients of monthly AMO index and six tree-ring chronologies (1856–2007).

AMO	PC1	Taksimo	Tschita	Mangui	Khotugn	Mengkeshan	Zhigansk
Average	**0.37**	**0.27**	**0.34**	**0.42**	**0.17**	**0.19**	0.08
Jan	**0.30**	**0.24**	**0.25**	**0.34**	0.15	**0.21**	0.07
Feb	**0.27**	**0.19**	**0.26**	**0.32**	0.11	**0.18**	0.05
Mar	**0.24**	**0.18**	**0.24**	**0.27**	0.10	0.11	0.03
Apr	**0.32**	**0.23**	**0.32**	**0.32**	0.15	0.11	0.04
May	**0.28**	**0.22**	**0.24**	**0.32**	0.11	0.09	0.06
Jun	**0.30**	**0.19**	**0.26**	**0.32**	**0.17**	0.13	0.10
Jul	**0.36**	**0.24**	**0.31**	**0.38**	**0.21**	**0.19**	**0.17**
Aug	**0.36**	**0.24**	**0.33**	**0.37**	**0.22**	0.16	**0.17**
Sep	**0.37**	**0.26**	**0.34**	**0.41**	**0.19**	**0.19**	0.12
Oct	**0.33**	**0.28**	**0.34**	**0.43**	0.07	**0.24**	0.06
Nov	**0.34**	**0.28**	**0.32**	**0.45**	0.12	**0.24**	0.02
Dec	**0.34**	**0.31**	**0.31**	**0.42**	0.10	**0.20**	0.03

Notes: Bolded values for significance at the 95% confidence level as tested by Pearson correlation.

**Figure 4:**
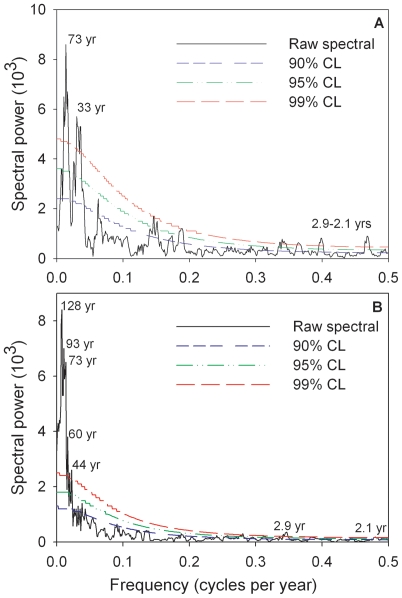
Multi-taper method spectrums for this proxy series from 1564–2007 (A) and the tree-ring AMO reconstruction from Atlantic rim [**20**] (B). Significance was tested at three levels (99%, 95% and 90%) against a red-noise background. Digital values are the significant periods at 99% confidence level.

**Figure 5:**
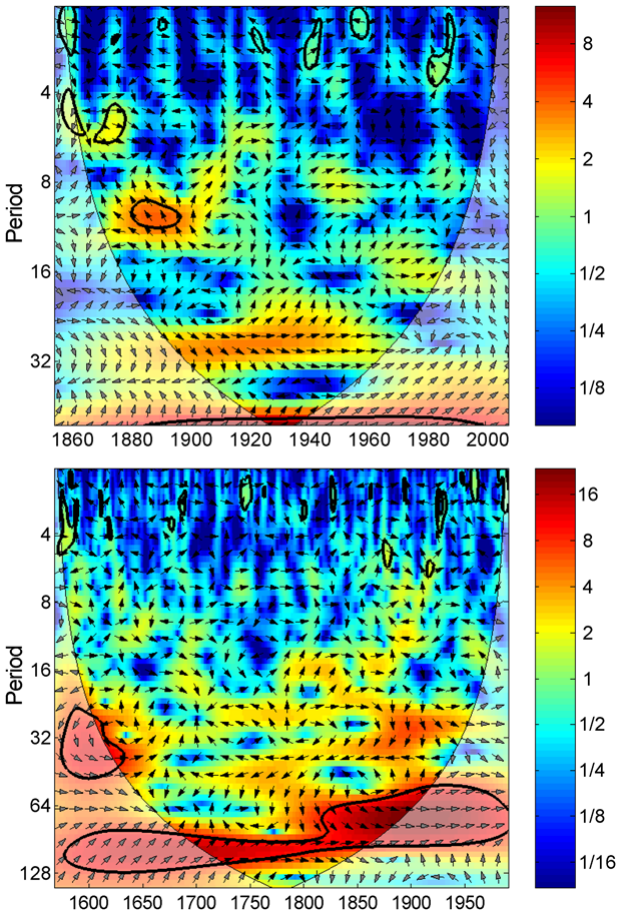
Cross wavelet transforms of this proxy series and the AMO index [**4**] (upper panel) and [**20**] tree-ring based AMO index (lower panel). The 95% significance level against red noise is shown as a black contour. The relative phase relationship is shown as arrows (with in-phase pointing right, anti-phase pointing left, and tree-ring index leading AMO by 90° pointing straight down).

MTM analysis of the East Asian PC1 series reveals pronounced variability at interannual (2.9–2.1 years) and multidecadal (33 and 73 years) time scales (Fig. 4A). The existing AMO reconstruction from the Atlantic rim [20] exhibited significant periods over a wide band from ∼40–128 year (Fig. 4B). However, the spectra for these two series showed very similar periodicities, with overlaps at 73, 2.9 and 2.1 years, while the AMO reconstruction from Atlantic rim has more long-periods such as 93, 60 and 44 year. A cross-wavelet transform of the East Asian PC1 series and the instrument AMO index shows a persistent and significant multidecadal signal centered on the roughly 70-year band almost throughout the whole instrumental period (1856–2007) (Fig. 5A). Cross-wavelet analysis tests the coherence between time series in the time-frequency domain. Similarly, the East Asian PC1 series also displays a strong coherence with the existing reconstruction in a band from ∼64–128 yr over the period 1568–1990 (Fig. 5B). These phase relationships indicate that the East Asian PC1 series and both the instrumental AMO index and the previous reconstruction are almost in phases in the sectors with significant common power. These also are confirmed by visual comparison of the series in Fig. 3. Additionally, there are short periods of significant common power in the 20- to 40- year bands between the two series from the late 1570s to the early 1620s. This may correspond to the shorter periodicity that [29] and [19] recently attributed to the AMO. MTM and cross-wavelet analysis support the probable link between this proxy series and multidecadal Atlantic SST variability.

## Discussion

Tree-ring time series from northeast Asia exhibit a coherent, AMO-like pattern of variability over the last 440 years. Although a mechanism for AMO influence on East Asian climate is not fully understood, findings reported here support other observational and modeling studies that have demonstrated multidecadal Asia monsoon variability is related to the AMO [15]–[17]. Winters tend to be warmer and wetter than normal in northeast China during positive phases of the AMO [17], which induces negative surface pressure anomalies across the North Atlantic to extend eastward to Eurasia and facilitate a weaker Mongolian High, and thus a subdued East Asian winter monsoon. Positive phases of AMO also are characterized by elevated air temperatures in East Asia and increased rainfall in northeast China in all four seasons [16]. These influences may arise from warming Eurasian middle and upper troposphere that result in weakened Asian winter monsoons but enhanced summer monsoons. All of these patterns together suggest that positive AMO phases may lead to moister conditions and longer growing seasons (warmer falls) in northeast Asia, which is supported by the comparisons of PDSI over the latest warm and cold AMO phases at six sampling sites (Table 3). An opposite pattern appears in negative phases of the AMO. In addition, precipitable water, a measure of moisture availability, is a total amount of water vapor in a column of the atmosphere, measured as if all fell to the ground as precipitation. It may affect temperature and precipitation, which in turn affects tree growth. Despite the low correlation coefficients, this point is also confirmed by spatial correlation fields between annual mean precipitable water and the instrumental AMO index (1948–2007) in this region (Fig. 1).

**Table 3:** Comparison of PDSI for the warm and cold AMO phases at six nearby sampling sites.

Site	PDSI Mean ± SD	
	1930–1960	1970–1990
Taksimo	0.09±1.52	−0.18±2.00
Tschita	0.19±2.07	0.07±2.53
Mangui	0.40±2.55	−0.12±2.52
Khotugn	−0.03±2.87	−0.94±1.64
Mengkeshan	0.14±2.36	−0.59±2.58
Zhigansk	0.12±2.33	−0.56±1.97

The results of correlation analyses between tree-ring chronologies and climatic factors indicate that both temperature and precipitation influence Scots pine growth at northeast Asia (not shown here), while the precipitation has less influence on tree-ring widths than temperature in this area especially for Khotugn and Mengkeshan. Also, the precipitation in the previous autumn and winter is important to tree growth, while the temperature have the similar influence during the previous autumn and current growing season. The cross correlation between the tree-ring record from northeast Asia and Gray et al. (2004) AMO reconstruction indicates that the maximum correlation (0.26) occurred at lag one year (Fig. 2). This may imply that the temperature or precipitation change of the AMO will affect the following tree growth in northeast Asia, or which may result from the strong first-order autocorrelation in this tree-ring record.

Further research is needed to explore how multidecadal variability in East Asian climate related to the AMO may have contingent effects on interannual to decadal climate variations and ecosystem processes. Recent studies of both Pacific and Atlantic Ocean climate teleconnections in western North America found that warm phases of the AMO synchronized sub-continental-scale patterns in droughts and forest fires across the western U.S [6], [30]. While both the El Nino-Southern Oscillation (ENSO) and Pacific Dedacal Oscillation (PDO) were the main drivers of interannual to decadal patterns in droughts and fires, AMO conditionally changed the strength and spatial influence of ENSO and PDO effects at multidecadal time scales.

This is the first Asian AMO-like proxy series spanning the last four centuries. The proxy series confirms and extends previous observational and modeled analyses in which it was found that the AMO plays a role in the multidecadal variability in East Asian climate. Furthermore, our results support that the AMO continues with its quasi-cycle of roughly 70 (65–80) years as well as shown in other proxy series [1], [19]–[21]. Thus, the present AMO proxy record has important implications for explaining past climate change and forecasting future climate anomalies in northeast Asia.
